# A customizable cost-effective design for printed circuit board-based nanolayered gold screen-printed electrode: From fabrication to bioapplications

**DOI:** 10.3389/fbioe.2022.1036224

**Published:** 2022-11-04

**Authors:** Sadegh Ghorbanzadeh, Seyed Morteza Naghib, Ali Sadr, Fatemeh Molaabasi, Wei Zhang

**Affiliations:** ^1^ State Key Laboratory of Structure Analysis for Industrial Equipment, Department of Engineering Mechanics, Dalian University of Technology, Dalian, China; ^2^ Nanotechnology Department, School of Advanced Technologies, Iran University of Science and Technology (IUST), Tehran, Iran; ^3^ Department of Electrical Engineering, Iran University of Science and Technology, Tehran, Iran; ^4^ Biomaterials and Tissue Engineering Research Group, Department of Interdisciplinary Technologies, Breast Cancer Research Center, Motamed Cancer Institute, ACECR, Tehran, Iran

**Keywords:** screen-printed electrodes, printed-circuit board, 2D printing, gold sputtering, biosensor (OB)

## Abstract

Screen-printed electrodes (SPEs) are promising candidates for fabricating biosensing platforms in the laboratory and industry due to the various advantages they involve. The primary method for fabricating SPEs is 2D printing. However, commercial SPEs have some limitations due to the specific ports and connections they require, inflexible design, high prices, and decreased efficiency after a short time. This article introduces high performance, feasible, and cost-effective gold SPEs based on the combination of printed circuit board substrate (PCBs) and sputtering methods for electrochemical biosensing platforms. First, we discuss a general gold SPE development procedure that helps researchers to develop specific designs. The final developed version of SPEs was characterized in the second step, showing positive performance in electrochemical parameters because of the optimization of design and fabrication steps. In the study’s final phase, SPEs were used to fabricate a simple platform for breast cancer cell detection as a proof of concept without using any linker or labeling step. The designed immunosensor is very simple and cost-effective, showing a linear calibration curve in the range of 10 − 2× 10^2^ cells mL^−1^ (*R* ^2^ = 0.985, S/N = 3). This research can be used as a reference for future studies in SPEs-based biosensors because of the flexibility of its design and the accessibility of the manufacturing equipment required.

## 1 Introduction

In recent years biosensing has become an attractive field in research and industry, so fabricating biosensors with high sensitivity and high efficiency in a fast and straightforward way is a critical goal for researchers (Naghib and Ghorbanzade, 2019). Several diagnostic and analytical devices and systems are currently used in many versatile applications such as food safety (Mishra et al., 2018) and the environmental (Hernandez-Vargas et al., 2018), and pharmaceutical industries (Cho, Kim, and Park, 2020), as well as clinical laboratories (Mathew et al., 2021). Most commercial point-of-care devices function with electrochemical sensors as an essential part. Over the last decade, SPEs with low cost, simple, and fast mass production has been used for emerging novel electrochemical biosensors (Pilas et al., 2019; Ghorbanzade, 2020; Huang et al., 2020) in the laboratory and in industry to improve performance using thick and thin-film technology. Due to their benefits, SPEs can handle some of the main challenges in biosensing platforms. They can also solve the sample size challenge in clinical applications due to their ability to miniaturize and integrate. Moreover, they have good potential for surface modifications. SPEs’ surface can be modified for versatile functions attributed to various analytes and reach various enhancements (Huang et al., 2019). SPEs help emeliorate several conventional drawbacks of common electrodes, such as tedious and time-consuming cleaning processes and memory effects (Banks, Foster, and Kadara, 2016).

Compared to the bulk layers, the fabrication process of nanostructured layers, the size, and the density of nanoparticles are considered more effective in electrical and chemical properties. Gold is an excellent material for creating nanostructured layers. The nanostructured electrode, made of AuNPs, is prepared by attaching AuNPs to the thiol spacer’s surface, which is self-assembled to a bulk electrode and the conductive substrate. The gold nanostructured electrodes can also be fabricated through *ex-situ* and *in situ* plating of the particles from the HAuCl_4_ solution on the substrate under a controlled circumstance, such as electro grafting (Raoof et al., 2010; Passos et al., 2015). The procedures mentioned above have some disadvantages, including 1) they are time-consuming, 2) they place demands on labo, 3) they require significant amounts of materials, and 4) a long professional user experience. In the gold nano-film electrodes produced by electro grafting, the thickness and the shape of the gold layers cannot be the same assuredly. Hence, the Au-nanostructured SPEs could be applied. To prepare the electrodes, ink is used on the substrate, usually produced by ceramic or plastic. Accordingly, the thickness of all the layers can be constantly measured. Moreover, compared to conventional bulk electrodes, these electrodes are cost-effective, meaning they can be used disposably for each measurement. It is noted that for fabricating the gold nano-film electrodes, the Au atoms would be deposited through physical vapor deposition using the sputtering method. The process can also be used for non-conventional reference electrodes or different arrangements of electrodes, which cannot be fabricated through the common screen-printed electrode production technique (Siegel et al., 2011). Sputtering is a preferred method because of its simplicity, reproducibility, pollution-free, and cost-effectiveness of the final product. However, the method has some demerits, for example, the adhesion of AuNP and the polymer substrate cannot be satisfied. Film adhesion is affected by the interface structure and changes when the electrode is exposed to some concentrated acids and organic solvents. The interface structure also affects the electrical properties of the electrodes. For this reason, the adhesion between the metal and the substrate is modified through some techniques, for instance using plasma treatments, fixation of the metal by thiol groups containing spacer, or increasing the substrate’s roughness (García-González et al., 2008) (Xiao et al., 2016) (Xiao et al., 2018).

Gold nano-film electrodes have advantages due to their particular physicochemical properties. Some of these advantages are as follows: change in its shape, easy miniaturization, possible change of the hydrophobicity or hydrophilicity, surface charge, and high surface-to-volume ratio. The properties can be improved through chemical modifications and electrochemical and mechanical pre-treatments. The electrodes can be also modified by adding other nanoparticles such as nanostructured carbon or metal. This combination results in different properties, a particular hybrid nanoparticle electrode with new characterization (Siegel et al., 2011). In addition to the electrode materials used in SPEs, the substrate plays an essential role in functioning. The electrodes are held in place by the substrate, which also serves as a connector. Due to their mechanical and electrical properties, printed circuit boards (PCBs) can be considered the building blocks of electronic devices. Up until recently, PCBs were used as substrates for single-layered circuits, but now they provide a substrate for fifty-layered circuits or more. The electrical components and connectors located on PCBs are linked *via* conductive copper routs to route electrical signals and power within and among devices (Dutta et al., 2019; Zhao et al., 2020). PCBs can therefore be utilized as SPEs substrates due to benefits such as reliability and cost-effectiveness (Liu et al., 2022).

**SCHEME 1:**
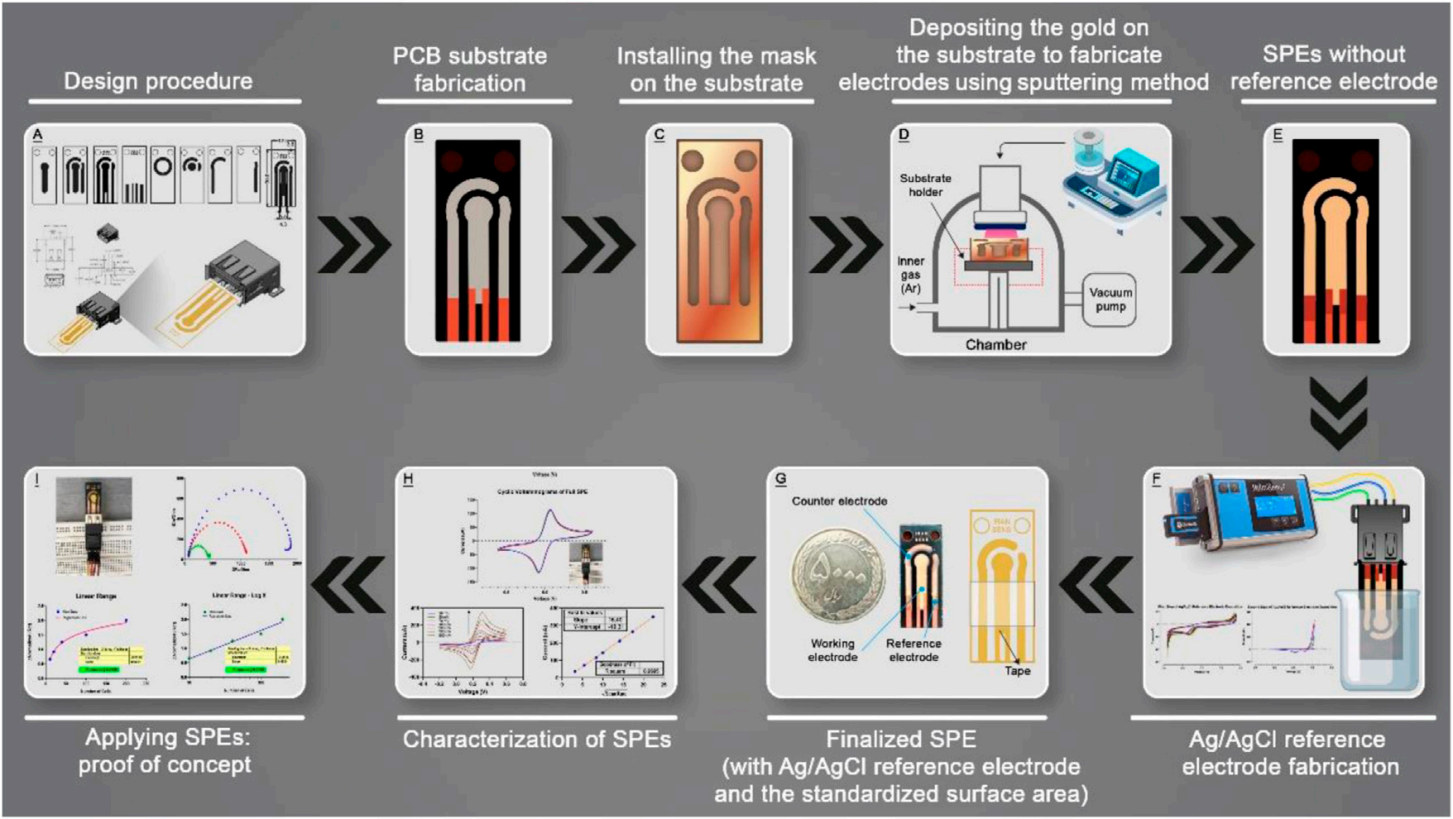
Schematic illustration of all steps of this study from designing to proof-of-concept test.

This study focused on the fabrication of screen-printed nanolayered gold electrodes based on first combining printed circuit boards and sputtering technology. In the next step, the characterization of screen-printed electrodes was investigated for electrochemical applications and finally, a simple immunosensor was fabricated and examined based on laboratory-made SPEs for proof of concept. All steps of this study are illustrated in Scheme 1.

## 2 Experiment

### 2.1 Chemicals

The gold sputtering target (99.999%), potassium ferrocyanide (K_4_Fe(CN)_6_), and potassium ferricyanide (K_3_Fe(CN)_6_) were provided by Sigma-Aldrich as the electrochemical probe. The electrochemical behavior of screen-printed electrodes in different supporting electrolytes was studied using sulfuric acid, potassium chloride, and potassium nitrate testing solutions. Bovine serum albumin (BSA) was purchased from Sigma Aldrich. Monoclonal anti-HER2 antibodies (AryoTrust product) from human resources were purchased from AryoGen Co-Iran. SK-BR3 cell line was obtained from Motamed Cancer Institute (MCI), Iran. All chemicals, materials, and reagents that were applied to prepare the buffer were exploited without further purification (analytical grade purity). Printed circuit boards were fabricated in Madar Sazan Novin Co (M.S.N), Iran.

### 2.2 Screen-printed electrode design

The design of the screen-printed electrode used in the project was undertaken according to the structure of commercial samples available on the market by Altium Designer 17 software. Because the purpose of this study was to design and build an electrode that could be customized and used in various projects and compatible with multiple devices, the structure design was developed based on the standard USB port so that the steps of making and using the port can be done quickly.

### 2.3 Fabrication of SPE substrate

After the design, the SPE substrate and copper connections were fabricated using printed circuit board fabrication technology in high-volume production capability according to the specifications listed in Table 1.

**TABLE 1:** Specifications of SPE printed circuit board substrate.

Parameter	Value
Substrate material	FR4
Substrate thickness	1.6 mm
Material of connections	Copper
Connection thickness	50 μm
Cutting method	V-Cut

### 2.4 Preparation of gold nanostructured film electrodes

After preparing the substrate (printed circuit board with connections) by placing the mask on the substrate, the gold deposition process was performed by the sputtering method. The parameters of the process were optimized, as listed in Table 2.

**TABLE 2:** Parameters of sputtering process for gold electrode fabrication.

Parameter	Value
Sputtering chamber pressure	70–80 mTorr
Power	50 W
Current	20 mA
Sputtering time	300 s
Environment after applying a vacuum	Argon
Repeat the sputtering process	1 or 2

### 2.5 Ag/AgCl solid-state pseudo reference electrode fabrication

This step was performed in two sub-stages. In the first step, a solution of 5 mM AgNO_3_ and 1 M KNO_3_ is provided. The electrode is immersed into the provided aqueous solution. The applied potential is swept from −0.9 to 0.9 V *versus* the reference electrode for 20 cycles (scan rate = 0.1 V s^−1^). The Voltammogram of the first step is shown in Figure 3. In the second step for chlorination, the electrode is dipped in a solution of 0.01 M HCl and 0.1 M KCl. The applied potential is swept from −0.15 to 1.05 V *versus* SCE for four cycles at a scan rate of 0.05 V s^−1^ (Lee et al., 2016).

### 2.6 Electrode surface refreshing

When screen-printed electrodes are kept for a long time on the shelf, as is sometimes needed, an electrochemical cleaning method can be used to refresh the surface of the working electrode. In this method, an aqueous solution of 0.1 M of H_2_SO_4_ is first prepared and then the screen-printed electrode is immersed in the solution. The potential is then swept from −0.4 to 1.4 V *versus* the reference electrode (Ag/AgCl) for 10 cycles (scan rate = 0.05 V s^−1^) (Adams, 1969).

### 2.7 Fabrication of the simple immunosensor

After the finalization of SPEs, the bare working electrode was suspended in monoclonal antibody solution (0.2 mg ml^−1^) overnight at 4°C under the static condition and rinsed with PBS1X solution to remove extra materials with non-specific adsorption. After that, the immunosensor is incubated in BSA solution for 5 min (5% m/v BSA—7°C—5% CO_2_ 95% Air) for blocking the uncovered surface area. Finally, the final prepared biosensor was rinsed with PBS solution three times (Salahandish et al., 2018).

### 2.8 Apparatus

The voltametric measurements were recorded by potentiostat PalmSens directed using PSTrace 4.8 software. The Ag/AgCl reference electrode with all the potential values was applied. The auxiliary electrode was the platinum wire.

Energy-dispersive X-ray spectroscopy (EDS, analyzer X-MaxN, 20 mm2 SDD detector, Oxford Instruments, United Kingdom) was used to assess the elemental composition. To explore the morphology of the prepared structure, a scanning electron microscope (SEM) and an atomic force microscope (AFM) were exploited. Accelerating voltages for SEM and SEM-EDS measurements were 2 kV and 10 kV, respectively. To prevent sample charging, aluminum conductive tape was used to attach all samples.

## 3 Results and discussion

### 3.1 Screen-printed electrode design development

**FIGURE 1:**
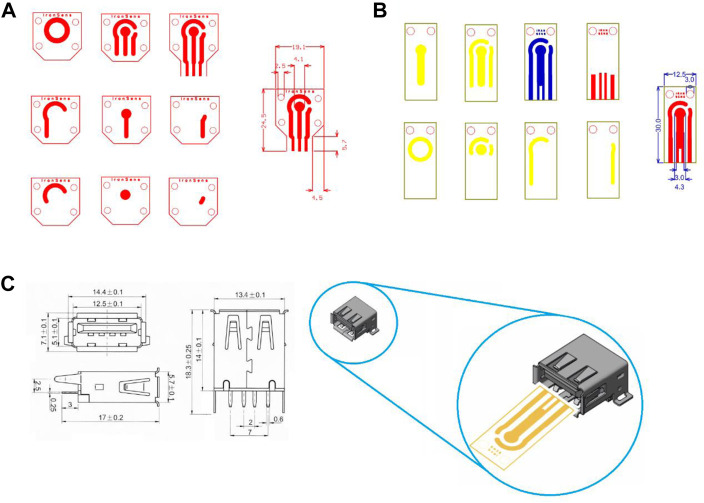
Screen printed electrode design **(A)** based on the port of PalmSens devices, **(B)** based on a standard USB port, **(C)** compatibility with USB port and dimension of USB port.

In SPE’s initial design, the number of electrodes and ports were designed to be compatible with the PalmSens3 device of Metrohm Company (Figure 1A). As shown in Figure 1A, due to the port limitations of the PalmSens3 device in the thickness of the strip substrate, as well as because the purpose of redesigning the strip was to make the general product customizable and usable in various projects, making it compatible with multiple devices. The design of the strip structure in the final version was modified based on the USB standard port (Figure 1B). Final design has been illustrated in Figure 1B and compatibility with the USB port could be observed in Figure 1C.

During the fabrication and use of the initial version of SPE, we found that the masks do not require four screws, so in addition to the changes related to the port design for ease to use, the physical and dimensional structure of the SPE, as well as the location of the slits for the mask screws, were designed to be more compact and practical. The SPEs are smaller and cheaper with two screws, thereby accelerating the next steps of the production process. In addition to the strip design, a set of applied masks was designed and made. They were used to complete and customize the SPE for making electrodes, reference electrode deposition, selective deposition, and so on.

**FIGURE 2:**
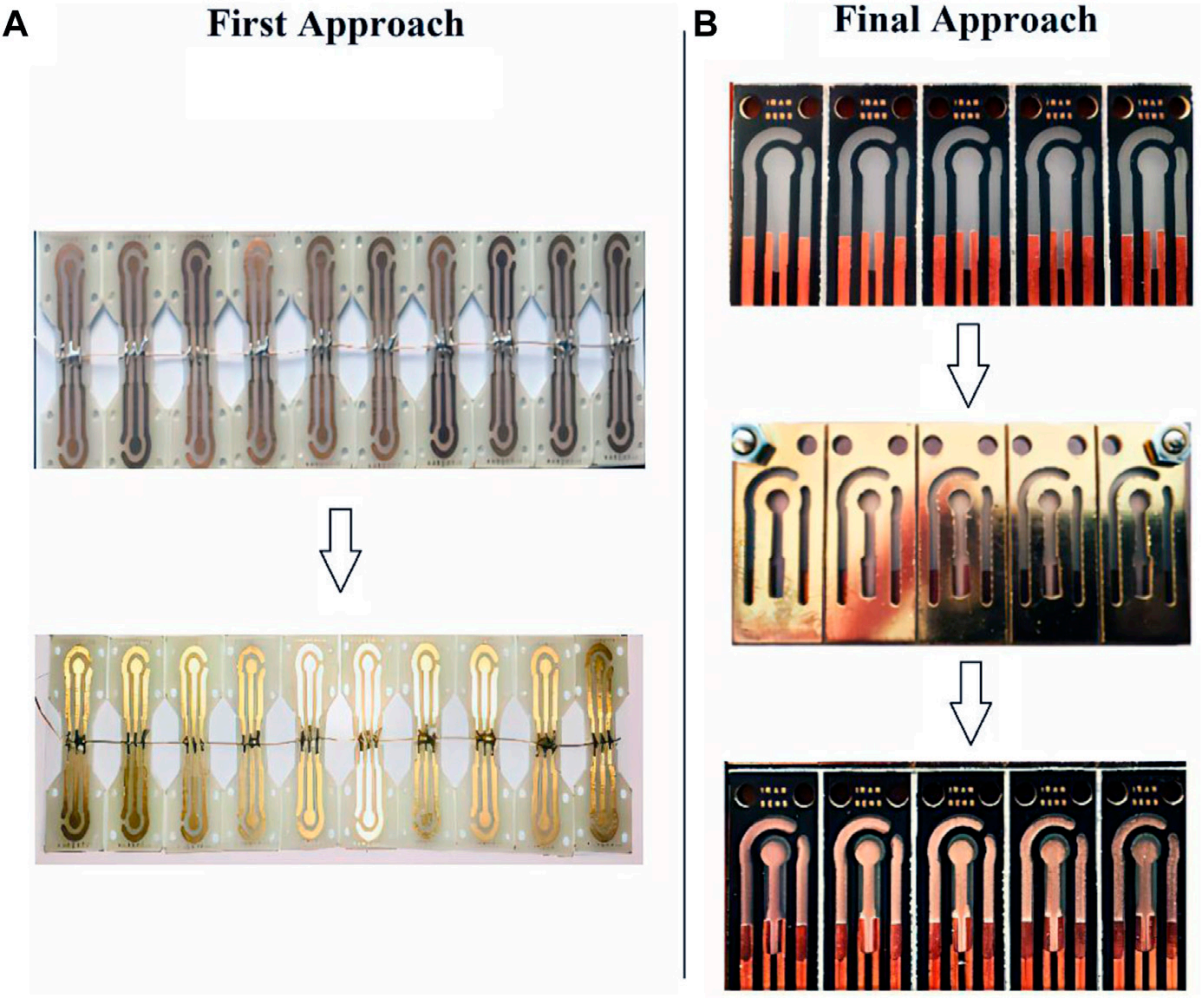
**(A)** First Approach Fabrication: Gold electroplating on copper electrodes in a two-step process, **(B)** Final Approach Fabrication: Gold sputtering on PCB substrate.

Two different approaches were used to make the strip electrodes: 1) by making copper connections and electrodes and then electroplating gold on copper and 2) by making copper connections and then using sputtering technology for electrode fabrication with designed masks. In the first approach, a complete strip is made of copper. The surface of the copper electrodes, a thin layer of gold is electroplated in a gold bath for 25 s (5 steps of 5 s) with a purity of 99.999% (Figure 2A). In the second approach known as the ‘final approach’, only connections that are in contact with the port and that do not come into contact with the solution are made of copper. After that, the electrodes are developed using the sputtering process (Figure 2B).

After initial investigations in the environment of electrochemical solutions to measure the voltage window of the electrode, it was found that the electrodes made with the first approach due to the presence of the copper substrate in the manufacturing process, have a minimal voltage window due to the copper oxidation voltage limit, which hinders many potential applications. Since the purpose of designing the SPE is to make a product with broad applicability, in the continuation of experiments, the second final manufacturing approach was used to make the SPEs. Since these SPEs have a wide and acceptable voltage window, this structure was the basis for the next steps of the study. In short, in the first approach, the fabrication process is easier and faster than the final approach but because of limited applications, its use was abandoned in this study’s next steps. However, the first approach can be used for non-laboratory applications with a predetermined voltage range located in the strip voltage window. According to the Zimmerpeacock and Drop Sense companies, the price of gold electrodes is around 2.5 to 4 euros (for mass purchase), which is 0.35 euros (for limited and lab-made production) for the electrodes presented in this study. A further benefit of the method presented in this study is its ability to customize the electrode design to fit different substrates and shapes for different applications, in addition to reducing the price.

### 3.2 Electrode characterization and evaluation

**FIGURE 3:**
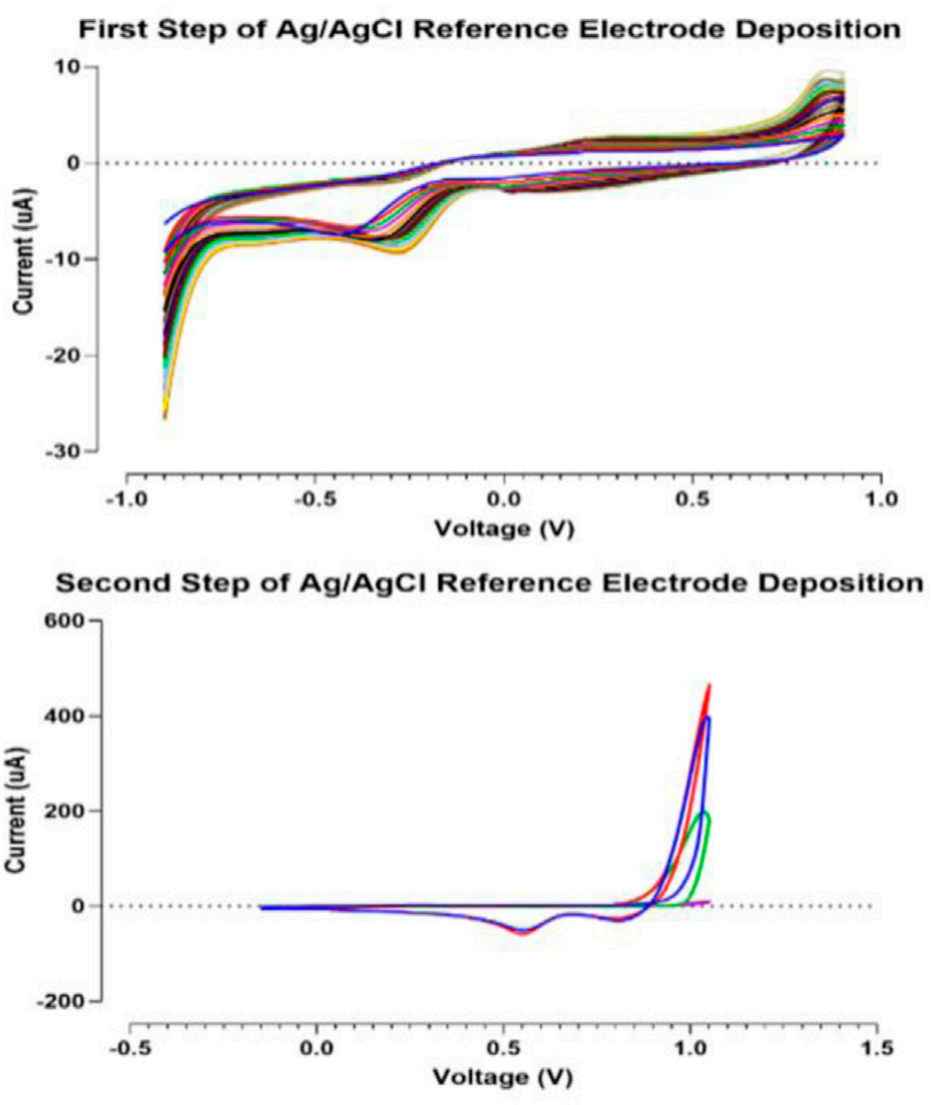
Voltammograms of Ag/AgCl reference electrode fabrication steps.

**FIGURE 4:**
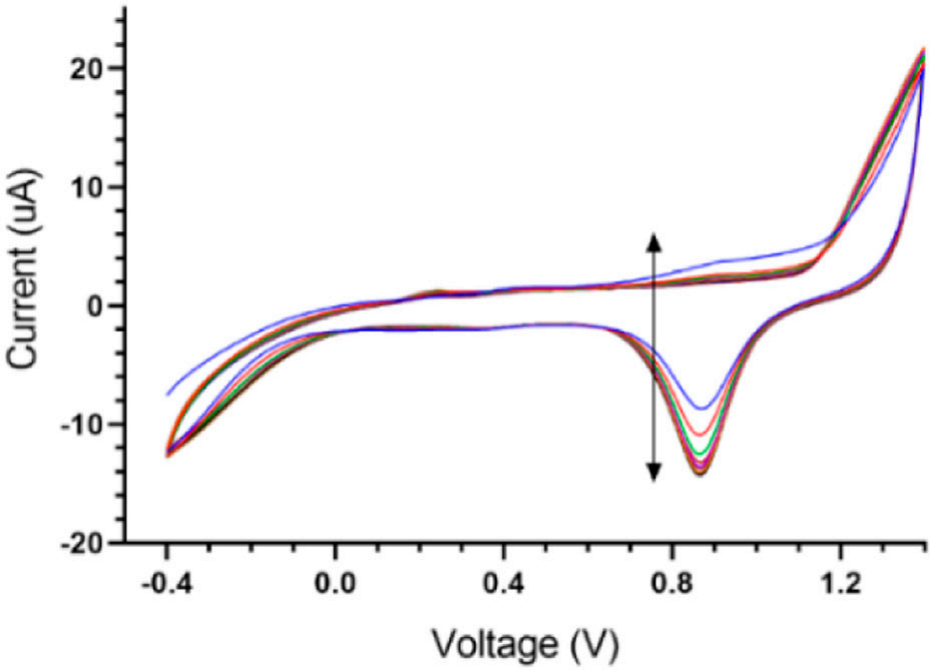
Cyclic Voltammograms of electrode cleaning in sulfuric acid.

In this section, data related to the fabrication and characterization process are summarized. The goal of this section is to ensure the repeatability of simple, cost-effective designed gold SPE so that the results can be reused in future research and comparisons. One of the steps that have a significant effect on the quality of SPE, is the fabrication of Ag/AgCl reference electrode, which is fully described in Section 2.5. of the experiment section. Figure 3 shows the voltammograms of this process step. Also, the electrochemical cleaning method could be used on the refreshing surface of the working electrode when novel designed screen-printed electrodes are kept for a long time on the shelf (2.6. section in experimental category). The related voltammograms are shown in Figure 4.

**FIGURE 5:**
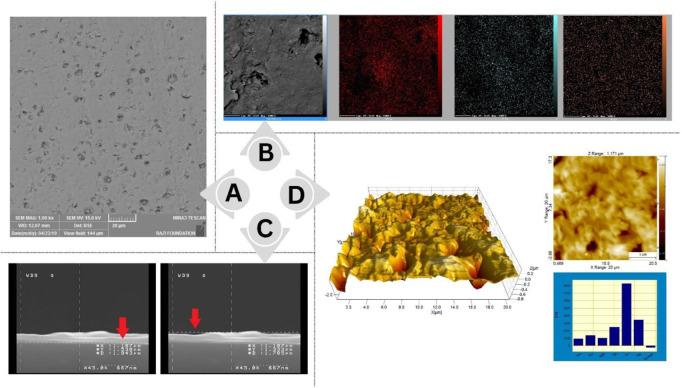
**(A)** SEM Scan of the surface of the gold SPE on a larger scale **(B)** SEM and EDX scan of the gold SPE’s surface **(C)** The SEM cross-section scan shows that the thickness of the electrode layer is in the range of 200 nm **(D)** AFM examination of the surface morphology of Au electrodes.

To evaluate the screen-printed electrodes, the chemical composition and morphology of the surface of fabricated gold nanostructured film electrodes were determined by SEM-EDS (Figures 5A,B). As shown in Figure 5C, the gold nanostructured film on the electrode surface is well demonstrated by the SEM results. The result of the AFM examination of the surface morphology of Au electrodes is illustrated in Figure 5D. The roughness value of the Au layer was calculated at approximately Ra = 100 nm and Sq = 135 nm. A rougher surface improves the gold layer’s adhesion to the substrate. The gold layer adheres well to the substrate based on the results of this study compared to previous studies (Libansky et al., 2017).

To explore the limitations related to the composition of electrolytes and interferences in the manufacturing process, the voltammetric behavior of the designed gold screen-printed electrode was tested in several supporting electrolytes including PBS buffers (pH 7.4), sulphuric acid, potassium nitrate, and potassium chloride. The outcomes were compared to the commercial gold bulk electrodes in previous works (Libansky et al., 2017); some of the outcomes were repeated for the gold screen-printed electrodes after 30 days of fabrication.

**FIGURE 6:**
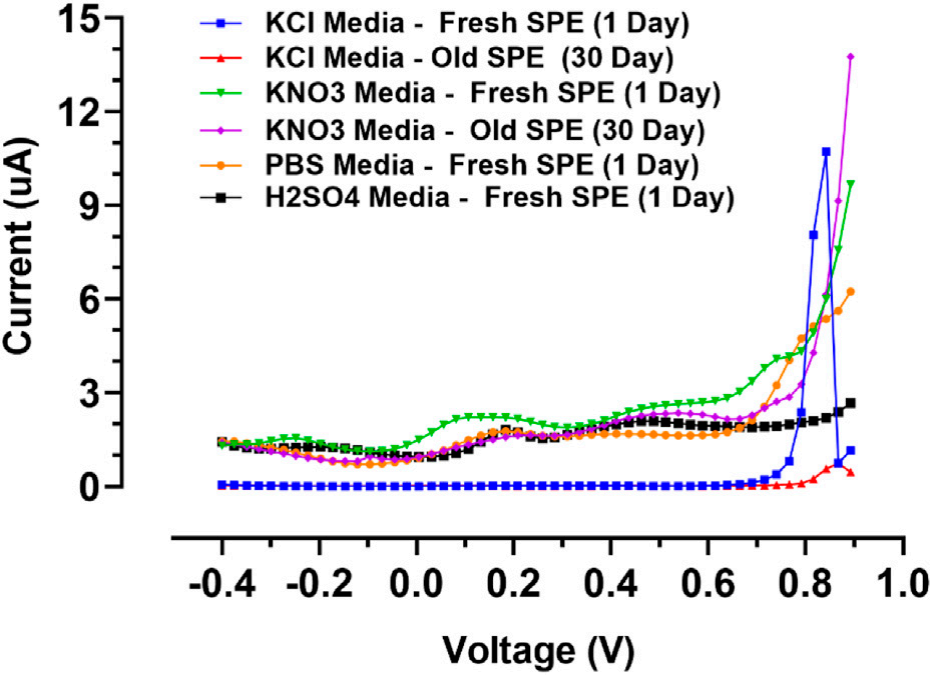
Differential pulse voltammograms of fresh and old SPEs in selected supporting electrodes.

The voltage window, which is summarized in Table 3; Figure 6, indicates the recorded DPVs in the anodic potential window. The electrochemical characteristics of the gold electrodes were realized in the acidic medium (H_2_SO_4_). The alpha and beta peaks of gold oxides in gold screen-printed electrodes were around 1.1 and 1.3 V, which was lower than those in gold bulk electrodes. This might be because the acidic environment disturbed the fine nanolayer of the electrode, resulting in the easier formation of gold oxides (Oesch and Janata, 1983). Potassium chloride and potassium nitrate were therefore selected as the proper media for voltametric nano-film electrode measurements. Here, the peaks of gold oxides combined with the end of the expansive potential window. The gold oxides can be removed in multiple ways: 1) by polishing the bulk electrodes, 2) by chemical cleaning using organic or inorganic solvents, and 3) by electrochemical cleaning with fast cyclic voltammetric scanning in the wide range of negative and positive potentials. In the case study in this article, the bulk electrode could be cleaned mechanically, while the gold nano-film commercial electrodes cannot be renewed mechanically because of their low mechanical robustness, they were utilized as disposable sensors. Notably, developed gold screen-printed nano-film electrodes can be renewed using CV electrochemical cleaning or chemical cleaning with concentrated ethanol. For electrolytes with neutral pH, electrochemical cleaning was used to prevent the reduction of thin nanolayer adhesion to PCB. Furthermore, potassium nitrate was used as an optimum medium because of the minimum background current in the wide working window.

**TABLE 3:** SPE’s voltage window in a common electrolyte.

Electrolyte type		Fresh electrodes (1 Day after fabrication)	Old electrodes (30 Days after fabrication)
Potassium Chloride	Min. voltage	−0.4	−0.4
	Max. voltage	0.8	0.8
Potassium Nitrate	Min. voltage	−0.4	−0.4
	Max. voltage	0.7	0.7
Sulfuric acid	Min. voltage	−0.4	—
	Max. voltage	0.9	—
PBS	Min. voltage	−0.4	—
	Max. voltage	0.7	—

**FIGURE 7:**
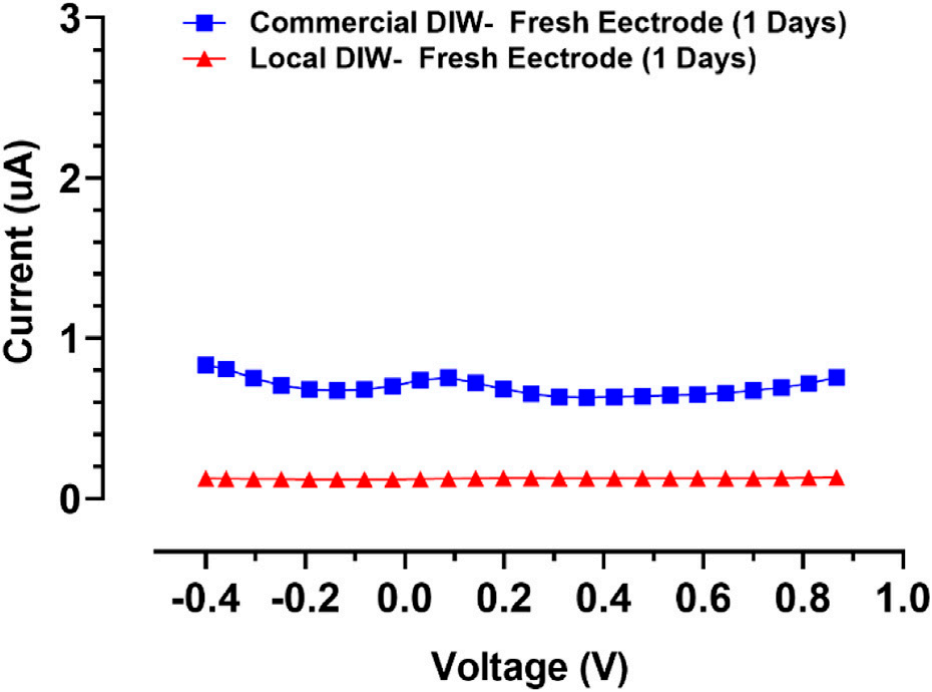
Differential pulse voltammograms for two types of DI for evaluation of fresh SPE’s sensitivity.

To evaluate the sensitivity of the behavior of the designed gold screen-printed electrode, differential pulse voltammograms for two types of deionized water (DI) were recorded, as shown in Figure 7. This test indicated that SPEs are very sensitive to the environment, showing a positive potential for accurate applications, meaning it could be used as a sensor for deionized water quality evaluation.

### 3.3 Electrochemical behavior of fabricated electrode

**FIGURE 8:**
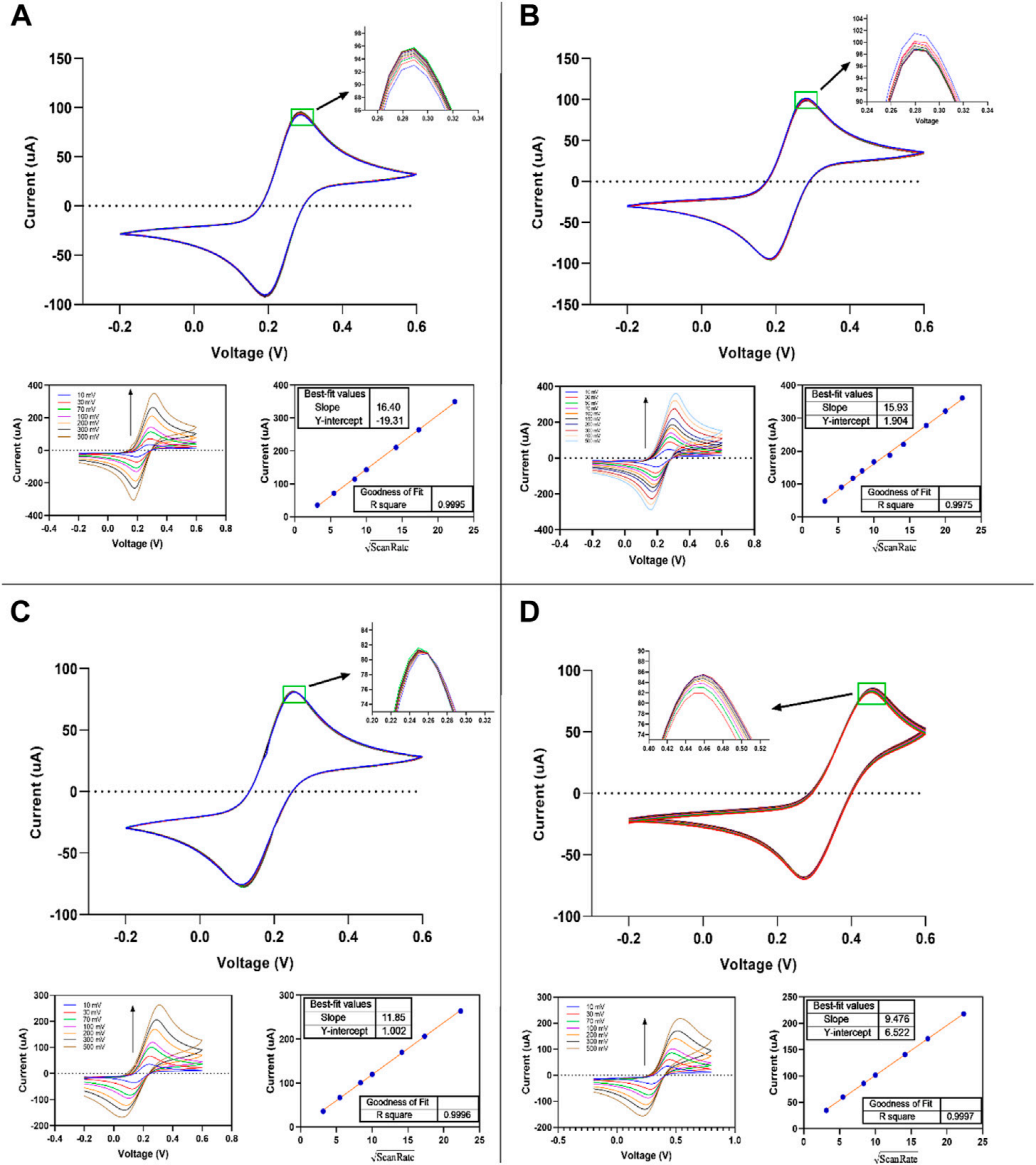
Cyclic voltammograms of fabricated thin-film gold SPE measured in 0.5 mM K_4_Fe(CN)_6_/K_3_Fe(CN)_6_ aqueous solution + **(A)** 1M KCl, **(B)** 1 M KNO_3_, **(C)** 1X PBS, and **(D)** 0.1 M H_2_SO_4_. Scan rate: 10–500 mV s^−1^.

The electrochemical characteristics of thin-film gold screen electrodes were explored using the cyclic voltammetry of ferricyanide/ferrocyanide redox in sulphuric acid, potassium nitrate, potassium chloride, and PBS buffers (pH 7.4). Fe(CN)_6_^3-/4-^ redox probe is most frequently used for characterizing the electrochemical aspects of the electrodes in aqueous solutions. The present study examined the dependence of the electrode response on the scan rate, reversibility, and repeatability of the measurements (Figure 8). The cyclic voltammograms of 5.0 mM Fe(CN)_6_^3-/4-^ at various scan rates were carried out by fabricated SPE in 0.1 M sulphuric acid, 1.0 M potassium nitrate, 1.0 M potassium chloride, and 1X PBS buffer (pH 7.4). The reversibility of the one-electron transfer reaction and the repeatability of measurements were determined from 10 cycles at 50 mV s ^−1^.

The values obtained of ∆Ep are slightly higher (∼10 mv) than that expected for theoretical Nernstian reversible one electron reaction (59 mV), indicating higher reversibility of the redox probe at the thin-film gold SPE. This has no interference with the use of designed SPEs in electrochemical biosensing and other electrochemical applications, so it is good and can be acceptable (Libansky et al., 2017). As a result, the anodic peak current values and the scan rate’s square root showed a linear good relationship (see Figures 8A–D). R Square coefficient affirmed the linearity of the obtained curves (∼1). The potassium chloride medium showed the highest and H_2_SO_4_ showed the lowest value in those slopes. All slopes in all media, especially in potassium chloride and potassium nitrate, are close to each other.

### 3.4 Fabrication repeatability and stability in response

**FIGURE 9:**
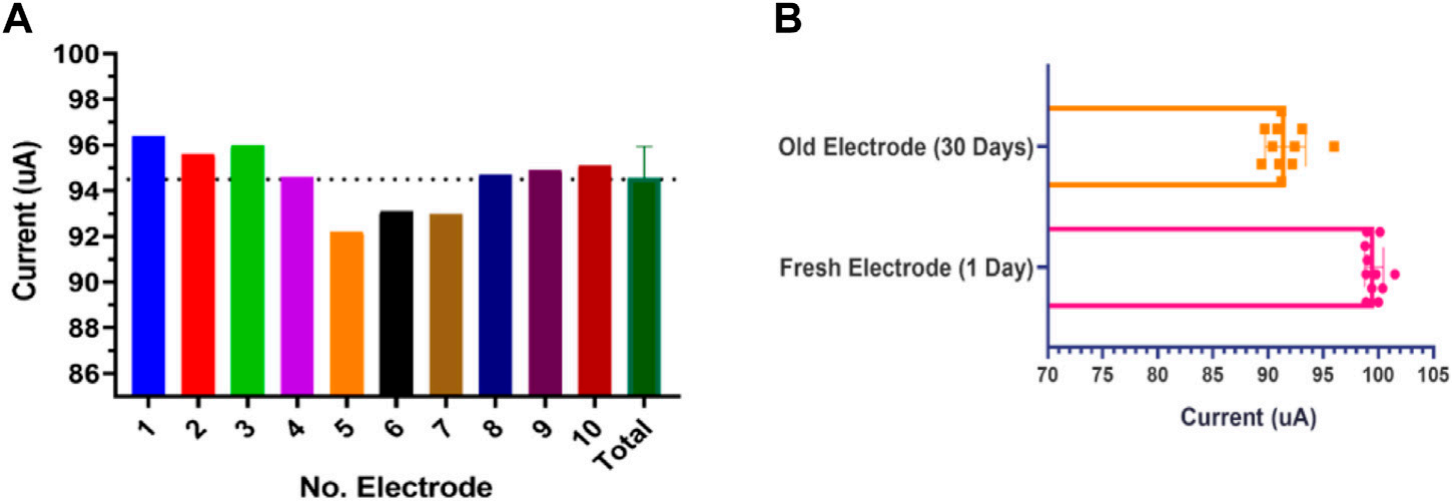
**(A)** Fabrication repeatability was examined for ten different SPEs based current response. **(B)** Response current stability for ten SPEs in 0.5 mM K_4_Fe(CN)_6_/K_3_Fe(CN)_6_ with 50 mV s^−1^ at 1 day and on the 30th day after fabrication.

The reproducibility of the electrode fabrication was evaluated by SPE current performance under the same laboratory condition for ten electrodes with three replications. As shown in Figure 9A the electrode current behavior for ten different electrodes in the same fabrication cycle provides good stability and little tolerance is observed in its behavior. As another essential parameter, the response stability of the fabricated SPE was evaluated. This parameter was examined for ten SPEs with three replications after 30 days. As you can see in Figure 9B, the strip has lost only 10% of its efficiency after 1 month in shelf storage conditions, indicating the perfect efficiency for the designed SPEs. This parameter was also investigated in storage space away from humidity and light for up to 3 months, and acceptable results were obtained. Altogether, it can be said that our novel fabricated thin-film gold SPE has satisfactory reproducibility and repeatability and retains its stability with time.

### 3.5 Effect of the internal and external reference electrode

**FIGURE 10:**
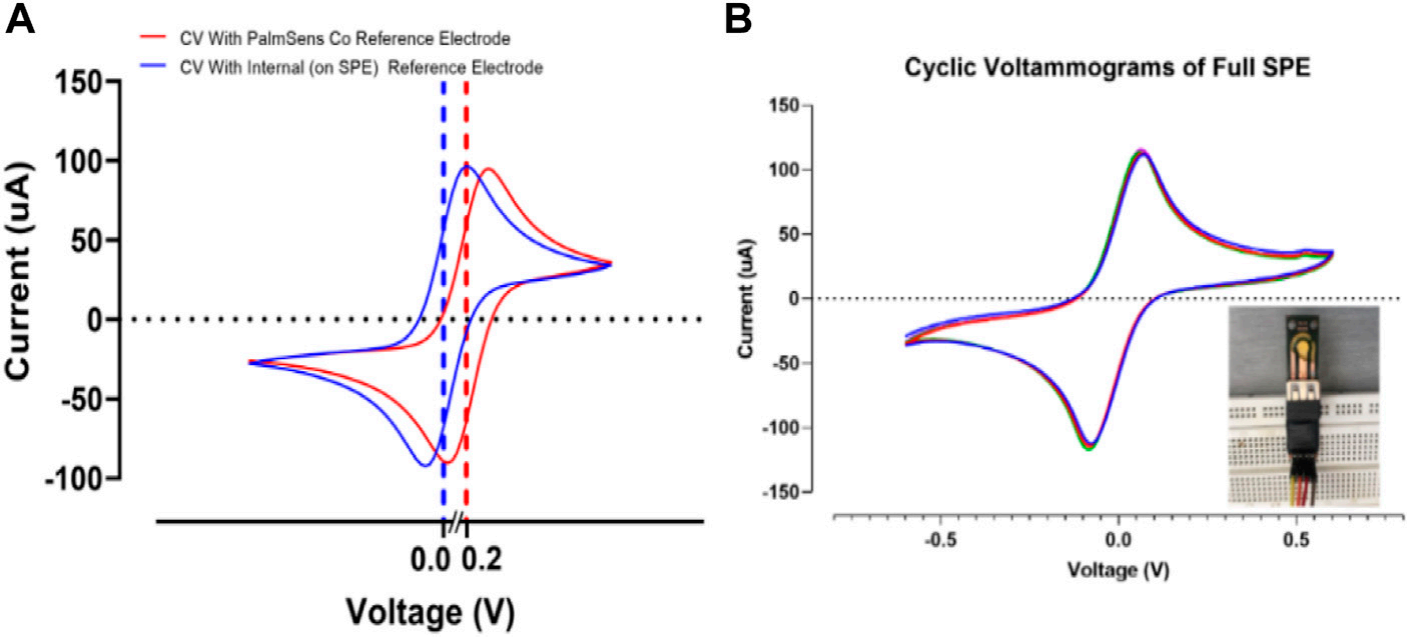
**(A)** Evaluating *the* effect of *the* internal and external reference electrodes on SPEs response. **(B)** Cyclic voltammograms of the full SPE with internal reference electrode for 10 cycles. All voltammograms were recorded in 0.5 mM K_4_Fe(CN)_6_/K_3_Fe(CN)_6_ at 50 mV s^−1^.

In this section, the effect of internal (deposited reference on the SPE) and commercial external reference were examined by cyclic voltammetry test (Figure 10). When the external reference electrode was used, a shift was only observed at the peak point of the diagram, and the peak structure was fully preserved, indicating the stable behavior of the strip. The observed shift shows the effect of minor voltage changes on the reference electrodes (Figure 10A). In the case of the internal reference electrode, as shown in Figure 10A, the structure of the peak is fully preserved and the peak is shifted around 0 V. This indicates the correct operation of the reference electrode with a few millivolts due to the thin layer of the reference electrode. This is confirmed in previous research (Libansky et al., 2017). Figure 10B demonstrates a good stability response in the full SPE with an internal reference electrode. After 10 cycles the current response in cyclic voltammograms showed insignificant changes, allowing the perfect stability of the developed PCB-based strips (Lee et al., 2016).

### 3.6 Performance evaluation of the fabricated SPE as a simple impedimetric immunosensor: Proof of concept

**FIGURE 11:**
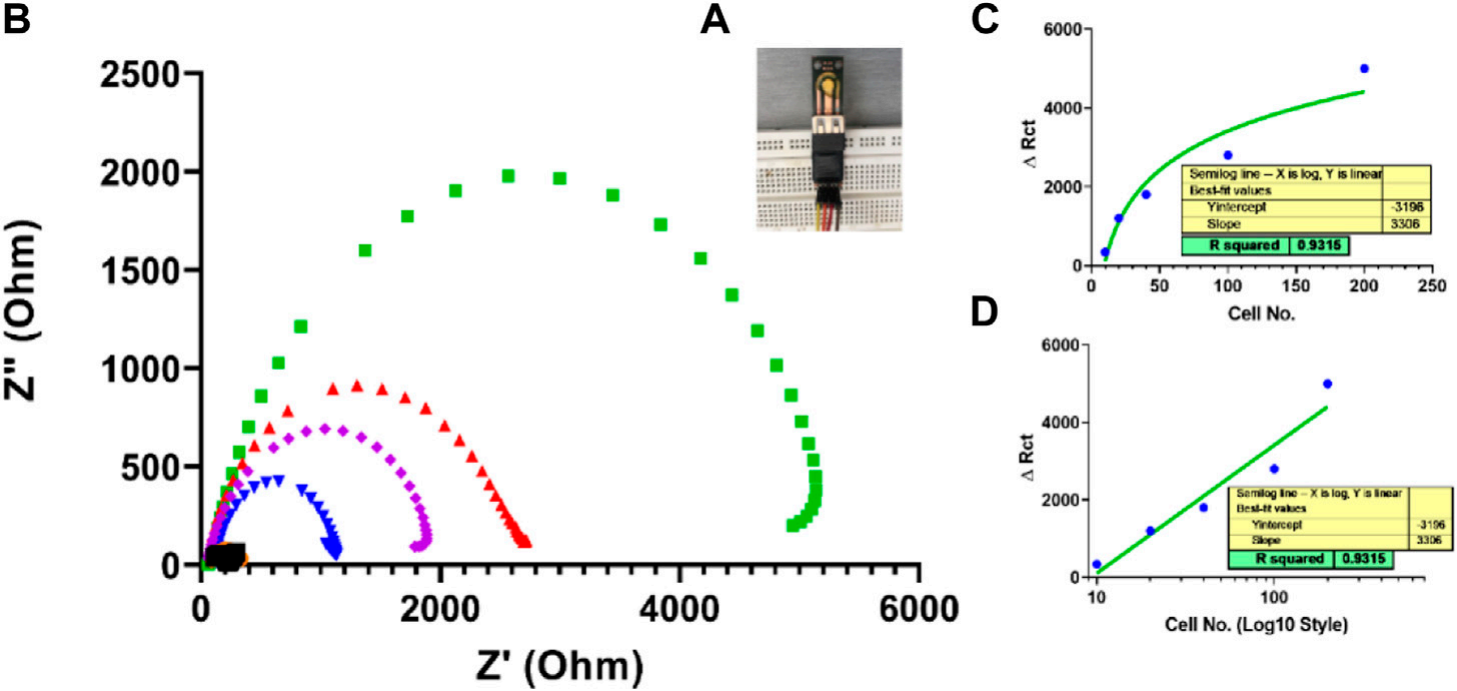
**(A)** A simple impedimetric immunosensor based on lab-made SPEs. **(B)** Electrochemical impedance measurements for three samples based on SPEs EIS response of Herceptin-modified thin-film gold SPEs toward different SKBR3 cell concentrations (10, cells mL^−1^) in PBS with 0.5 mM [Fe(CN)_6_]^3−/4−^. **(C)** Calibration curve for log C_cell mL_^−1^ *versus* R_ct_ from 10 to 200 cellsmL^−1^ Linear range of fabricated simple immunosensor for 10 to 200 cells. **(D)** Logarithmic linear range of the fabricated simple immunosensor for 10 to 200 cells.

After fabricating and characterizing the PCB-based gold strips as the first practical step to confirm bioimpedance measurement applicability, the coast-effective fabricated SPEs were used as a diagnostic biosensor for SKBR3 human breast cancer cells without any special surface modification. This novel fabricated electrode was designed simply, only by a drop casting 10 µl of the biological recognition element Herceptin monoclonal antibody on the surface of the unmodified gold electrode. As seen in Figure 11, the charge transfer resistance (R_ct_) increased with SKBR3 cell concentration from 10 to 200 cells, so that a linear range was obtained between ΔR_ct_ and the log concentration of the SKBR3 cells (*R* ^2^ = 0.9854) with a detection limit (LOD) of x cells mL^−1^ (S/N = 3). Figure 11D is another representation of Figure 11C. In all sensors, the behavior at the end of the linear response tends to be saturated, with the linear response shown in Figure 11C. As seen in picture 12D, by changing the scale of the graph to a logarithmic scale, the behavior was further investigated.

## 4 Conclusion

Gold micro/nano-layers prepared by sputtering technique (physical vapor deposition) using pure gold are usually employed for many biomedical and industrial applications. Herein, we focused for the first time on the fabrication of SPE based on combining a printed circuit board and sputtering technology. Then, the gold nanostructured film-sputtered SPE was characterized and evaluated for electrochemical purposes. To characterize the fabricated electrodes and reaction of standard redox probes (benzoquinone/hydroquinone and ferricyanide/ferrocyanide) SEM, EDS, and electrochemical tests were used for the analysis of morphology, chemical compositions, and electrochemical characteristics of the fabricated electrode. The evaluations of the electrodes were supplemented by the calculation of their real surface areas using the Randles-Sevcik equation. Finally, a simple immunosensor for cancer detection was fabricated and examined based on lab-made SPE for proof of concept. The impedance technique was then established for various samples and a linear range of data obtained. This immunosensor could detect 10 to 200 cells in the laboratory using HER2-positive breast cancer cells SKBR3 *in vitro*.

## Data Availability

The original contributions presented in the study are included in the article/Supplementary Material, further inquiries can be directed to the corresponding author.
